# Regulation of Mating-Induced Increase in Female Germline Stem Cells in the Fruit Fly *Drosophila melanogaster*

**DOI:** 10.3389/fphys.2021.785435

**Published:** 2021-12-07

**Authors:** Ryo Hoshino, Ryusuke Niwa

**Affiliations:** ^1^Degree Programs in Life and Earth Sciences, Graduate School of Science and Technology, University of Tsukuba, Tsukuba, Japan; ^2^Life Science Center for Survival Dynamics, Tsukuba Advanced Research Alliance (TARA), University of Tsukuba, Tsukuba, Japan

**Keywords:** germline stem cell, interorgan communication, mating, ecdysone, neuropeptide F (NPF), octopamine

## Abstract

In many insect species, mating stimuli can lead to changes in various behavioral and physiological responses, including feeding, mating refusal, egg-laying behavior, energy demand, and organ remodeling, which are collectively known as the post-mating response. Recently, an increase in germline stem cells (GSCs) has been identified as a new post-mating response in both males and females of the fruit fly, *Drosophila melanogaster*. We have extensively studied mating-induced increase in female GSCs of *D. melanogaster* at the molecular, cellular, and systemic levels. After mating, the male seminal fluid peptide [e.g. sex peptide (SP)] is transferred to the female uterus. This is followed by binding to the sex peptide receptor (SPR), which evokes post-mating responses, including increase in number of female GSCs. Downstream of SP-SPR signaling, the following three hormones and neurotransmitters have been found to act on female GSC niche cells to regulate mating-induced increase in female GSCs: (1) neuropeptide F, a peptide hormone produced in enteroendocrine cells; (2) octopamine, a monoaminergic neurotransmitter synthesized in ovary-projecting neurons; and (3) ecdysone, a steroid hormone produced in ovarian follicular cells. These humoral factors are secreted from each organ and are received by ovarian somatic cells and regulate the strength of niche signaling in female GSCs. This review provides an overview of the latest findings on the inter-organ relationship to regulate mating-induced female GSC increase in *D. melanogaster* as a model. We also discuss the remaining issues that should be addressed in the future.

## Introduction

In many animal species, mating not only transfers sperms to females, but also induces different behavioral and physiological changes that are known as the post-mating response; this response has been reported in both vertebrates and invertebrates. For example, in animals that do not have an estrus cycle (so-called induced ovulators such as rabbits, cats, and minks), mating stimuli can induce the formation of a follicular cavity and then ovulation (Bakker and Baum, 2000). In some female moths, such as the corn earworm *Helicoverpa zea*, the old world bollworm *Helicoverpa armigera*, and the silkworm *Bombyx mori*, the secretion of sex pheromones to attract males decreases after mating, which is followed by termination of calling behavior and initiation of oviposition behavior (Bali et al., 1996; Rafaeli, 2002; Nagalakshmi et al., 2007).

In the last several decades, many researchers have been interested in how post-mating responses are evoked by mating stimuli at the molecular and cellular levels. To elucidate the mechanisms, the fruit fly *Drosophila melanogaster* has served as the most powerful genetic model organism. One of the monumental works of *D. melanogaster* post-mating responses is the identification and characterization of the male seminal fluid peptide: sex peptide (SP) (Chen et al., 1988; Chapman et al., 2003; Liu and Kubli, 2003); SP is a 36-amino-acid length peptide transferred from a male to a female during mating (Chen et al., 1988; Heifetz et al., 2001; Bloch Qazi et al., 2003; Liu and Kubli, 2003; Krupp and Levine, 2014). After SP is injected into the female uterus, it is received by a G-protein coupled receptor: SP receptor (SPR) (Yapici et al., 2007; Häsemeyer et al., 2009). SPR-positive sensory neurons (SPSNs), whose cell bodies are located near the uterus, project to the central nervous system and trigger a series of post-mating responses in females (Feng et al., 2014). SP is essential for most of the known post-mating responses in *D. melanogaster*, such as food intake, diet preference, refusal to mate, egg-laying behavior, organ remodeling, and oogenesis (Kubli, 2003; Ribeiro and Dickson, 2010; Krupp and Levine, 2014; Reiff et al., 2015; Walker et al., 2015; White et al., 2021a).

Oogenesis in *D. melanogaster* adults is initiated from female germline stem cells (GSCs), which are capable of self-renewal and differentiation into gametes. To ensure a steady supply of oocytes, the balance between self-renewal and differentiation in female GSCs must be meticulously maintained (Xie and Spradling, 2000; Drummond-Barbosa and Spradling, 2001). Dysregulated self-renewal and inappropriate differentiation of GSCs lead to gradual loss of GSCs, where the organisms are unable to form oocytes (Xie and Spradling, 1998). Like various types of stem cells in diverse organisms, the precise balance of self-renewal and differentiation in *D. melanogaster* GSCs is modulated by a microenvironment surrounding the stem cells, called a niche. The niche is usually composed of somatic cells and an extracellular matrix. *D. melanogaster* GSCs maintain their stemness by receiving juxtacrine and/or paracrine factors derived from the niche, such as Decapentaplegic (Dpp), Wnts, Hedgehog, Unpaired, and possibly Netrin (Hayashi et al., 2020; Zhang and Cai, 2020; Tu et al., 2021; Yatsenko and Shcherbata, 2021). Although many studies have reported that the niche signals are critical in regulating the proliferation and maintenance of GSCs, whether and how the number of GSCs is regulated by the external cues, such as mating, remains elusive. Since mating utilizes numerous eggs, it seems plausible that it would be one of the primary factors influencing the number of female GSCs.

Indeed, in 2016, our group reported a novel SP-mediated post-mating response in *D. melanogaster*, which is a mating-induced increase in female GSCs (Ameku and Niwa, 2016; Yoshinari et al., 2019). Since the discovery of post-mating increase in female GSCs, our group has intensively studied how SP-mediated mating information can affect the increase in number of female GSCs. We have successfully identified essential neuroendocrine factors, such as steroid hormones, neuropeptides, and monoaminergic neurotransmitters (Ameku and Niwa, 2016; Ameku et al., 2018; Yoshinari et al., 2020). In this review, we focus on the role of the neuroendocrine system in transmitting mating stimuli to female GSCs and discuss the future direction of our research.

## Female Germline Stem Cells in *Drosophila Melanogaster*

**FIGURE 1:**
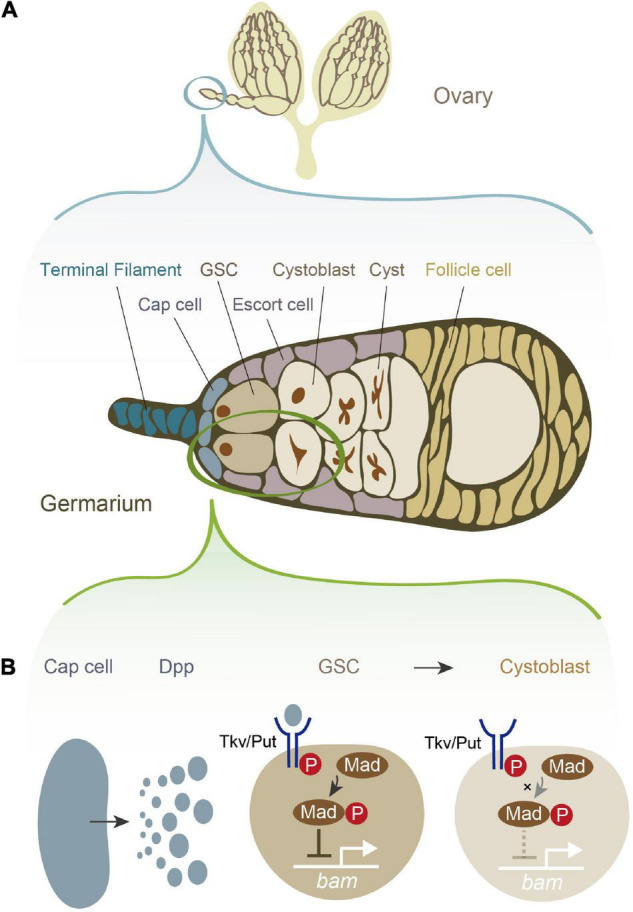
Female germline stem cells (GSCs) and their niche signal in *Drosophila melanogaster.* **(A)** A schematic illustration of *D. melanogaster* ovary and germarium. The germarium is located at the anterior tip of the ovary. The germarium consists of both germ cells and somatic cells. Germ cells contain female GSCs, cystoblasts, cysts, and more differentiated oocytes. Somatic cells contain terminal filament cells, cap cells, escort cells, and follicle cells. While all of these somatic cells contribute to the niche in some degree, the most important source of the niche signal is thought to be cap cells. **(B)** The niche signal from cap cells. Cap cells secrete Decapentaplegic (Dpp). Dpp is received by its receptor, a heterodimer of Thick vein (Tkv) and Punt (Put). The activated Dpp signaling eventually phosphorylates the signal transduction molecule Mother-against-dpp (Mad). The phosphorylated Mad (pMad) transcriptionally represses *bag-of-marbles* (*bam*). As *bam* is essential for promoting the cystoblast differentiation from female GSCs, the repression of *bam* is conversely essential for maintaining stemness.

*Drosophila melanogaster* female GSCs have been utilized as a powerful model system to study the role of niche signals in the self-renewal and differentiation of stem cells, as it is relatively easy to anatomically visualize and genetically manipulate GSCs and their niche (Ables and Drummond-Barbosa, 2010; Greenspan et al., 2015; Hayashi et al., 2020; Hinnant et al., 2020; Lin and Hsu, 2020; Zhang and Cai, 2020). Female *D. melanogaster* has a pair of ovaries, each of which is composed of approximately 16 ovarioles. Each ovariole possesses one to three GSCs at its germaria, the anterior tip of the ovariole (Figure 1A; Greenspan et al., 2015). At the periphery of the female GSCs, several types of somatic cells contribute to the niche, including cap cells, terminal filament cells, and escort cells (Figure 1A). Among these somatic cells, the cap cells, which are adjacent to the female GSCs, play a pivotal role in maintaining the balance between self-renewal and differentiation. Among the multiple niche signals including several juxtacrine/paracrine factors that the cap cells release (Hayashi et al., 2020; Zhang and Cai, 2020; Tu et al., 2021; Yatsenko and Shcherbata, 2021), Decapentaplegic (Dpp), a homolog of mammalian bone morphogenic protein (BMP), is particularly crucial for regulating female GSC stemness (Figure 1B; Xie and Spradling, 1998; Kai and Spradling, 2003). Loss of function mutations in *dpp* accelerates GSC loss and retard GSC division, while overexpression of *dpp* leads to GSC tumors (Xie and Spradling, 1998). Dpp is received by neighboring female GSCs, and it promotes the phosphorylation of the Dpp signaling mediator Mother against dpp (pMad). pMad and its downstream molecules eventually suppress the expression of *bag-of-marble* (*bam*), which is required for the transition from GSCs to more differentiated germ cells (Figure 1B; Chen and McKearin, 2003). Consequently, *bam*-negative GSCs maintain an undifferentiated state and maintain stemness. Conversely, cells that are physically separated from the cap cells receive no or low levels of Dpp, allowing the cells to express *bam* and then differentiate into cystoblasts (Hayashi et al., 2020).

## Mating-Induced Increase in Germline Stem Cells

As mentioned above, our group has reported that mating stimuli induce increase in female GSCs, which was contradictory to a previous report arguing that mating does not affect female GSC maintenance or division (Kao et al., 2015) (see section “Do Cryptic Genetic or Environmental Factors Induce Increase in Post-mating Increase in Germline Stem Cells?” for discussion). Each germarium has approximately 2.0 GSCs on average. However, beyond 16 h post-mating, the germaria containing 3 GSCs increases, resulting in an average number of approximately 2.5 GSCs in the germaria (Ameku and Niwa, 2016). This increase in the number of female GSC is sustained for 6 days, and lost after 7 days post-mating. This phenomenon is consistent with the effect of male seminal fluid on post-mating responses (Ameku and Niwa, 2016). Although this minute increase in female GSC numbers, from 2.0 to 2.5, may seem minimal, the overall number of GSCs increases from 32 in each ovary before mating to 40 GSCs after mating (assuming 16 ovarioles per ovary). Moreover, the number of cells seems to be directly reflected in the number of eggs laid in a week after mating. For example, as we describe later, in animals with impaired mating-induced GSC increase, by using RNAi-mediated knockdown of *neuropeptide F* (*NPF*), or a gene encoding an ecdysteroid biosynthesis enzyme, the number of laid eggs after mating is significantly lower compared to the animals proficient in mating-inducible GSC increase (Ameku and Niwa, 2016; Ameku et al., 2018). These findings suggest a positive correlation between the number of female GSC and the number of eggs laid.

It is also established that female GSCs increase in both M-phase and S-phase, suggesting that their division frequency is also accelerated (Ameku and Niwa, 2016). In addition, the intensity of pMad in female GSCs also increases after mating. However, no difference is observed in the number of cap cells before and after mating (Ameku and Niwa, 2016). In other words, the mating stimulus does not change the size of the GSC niche but induces an increase in female GSCs by strengthening the niche signal.

Similar to other post-mating responses, post-mating increase in female GSCs is mediated by the transfer of SP from males. For example, increase in female GSCs can be artificially induced by the overexpression of the *SP* gene in virgin females. In contrast, post-mating-induced increase in female GSCs can be suppressed by knocking down *SPR* specifically in SPSNs (Ameku and Niwa, 2016). These results indicate that the SP-SPR signaling pathway is necessary and sufficient for the increase in female GSCs.

Mating-induced increase in female GSCs can be speculated as an adaptive response to optimize the efficiency of egg formation in females. An adult female *D. melanogaster* may lay up to 100 eggs a day (Cohet and David, 1978). Assuming that the weight of a *D. melanogaster* female and an egg are approximately 1.2 mg (Okamoto et al., 2013) and 10 μg (Vijendravarma et al., 2010), respectively, it may be speculated that an adult female *D. melanogaster* possibly needs to produce those number of eggs in 1 day whose total weight is almost equivalent to the female’s body weight. Therefore, the process of egg formation is very costly for the females. If sexually reproducing insects can link fertilization and egg formation, it will be an effective strategy to increase the efficiency of offspring production. Therefore, the increase in GSCs during mating is considered an important process for activating fertilization and egg formation.

Of note, a recent study reported that mating stimuli alter the dynamics of male GSCs in *D. melanogaster* (Aston and Schulz, 2020; Malpe et al., 2020). Unlike female GSCs, there is no significant change in the number of male GSCs before and after mating. However, the number of male GSCs increase after mating during mitosis (Aston and Schulz, 2020; Malpe et al., 2020), which is similar to what we observe in post-mating female GSCs (Ameku and Niwa, 2016). To date, it remains unclear whether mating-induced male GSC mitosis is mediated by SP-SPR signaling. RNAi screen has identified several G-protein coupled receptors as potential candidates for mating-induced male GSC mitosis (Aston and Schulz, 2020; Malpe et al., 2020). It would be intriguing to examine how common and diversified are the molecular and cellular mechanisms of mating-induced GSC mitosis between males and females. Additionally, as spermatogonial stem cells are regulated by neuroendocrine factors such luteinizing hormone and testosterone (Kanatsu-Shinohara et al., 2004; Tanaka et al., 2016), studies on neuroendocrine system regulating male *D. melanogaster* GSCs would be interesting and useful from a comparative perspective.

## Humoral Factors that Transmit Mating Stimuli to Female Germline Stem Cells

**FIGURE 2:**
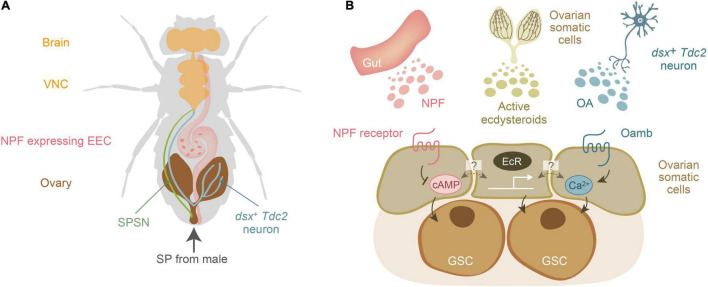
Three humoral factors essential for mating-induced increase in female germline stem cells of *Drosophila melanogaster*. **(A)** A schematic representation of the anatomical position of the brain, ventral nerve chord (VNC), sex peptide receptor-positive sensory neuron (SPSNs), the ovary-projecting *dsx* + *Tdc2* neuron, the ovary, and the NPF-expressing enteroendocrine cell (EEC). Sex peptide (SP) from male seminal fluid is transferred to the female uterus. **(B)** Three humoral factors, ecdysteroids, neuropeptide F (NPF), and octopamine (OA), are released from the gut, ovary, and a subset of octopaminergic neurons (*dsx^+^ Tdc2* neurons), respectively. The dark colored lines in the ovary represents the ovarian somatic cells, which are the source of ovarian ecdysteroids. Active ecdysteroids (such as 20-hydroxyecdysone) are received by the nuclear receptors EcR. On the other hand, NPF and OA are received by their specific receptors belonging to the G-protein coupled receptor family. The second messengers for the NPF receptor and octopamine receptor (Oamb) are cAMP and Ca^2+^, respectively. Of note, it is currently unclear whether EcR, NPF receptor, and Oamb are expressed in the same ovarian somatic cells or whether each of them is expressed in distinct cell types of the ovarian somatic cells.

Notably, SPSNs project to the central nervous system, but not directly to the ovary (Feng et al., 2014; Wang et al., 2020), suggesting that SP-mediated mating-inducible increase in female GSCs cannot solely be explained by SPSNs. Therefore, it is expected that humoral factors, such as hormones and neurotransmitters, may also contribute to the mating-induced increase in female GSCs. After our genetic approaches (Ameku and Niwa, 2016; Ameku et al., 2017, 2018; Yoshinari et al., 2019, 2020), the three humoral factors have been identified: ecdysteroids, NPF, and octopamine (OA), originating from the ovary itself, the gut, and the central nervous system, respectively (Figures 2A,B). These factors are received by the GSC niche, and downstream signaling is essential for the increase in female GSCs and their maintenance.

### Regulation of Germline Stem Cells by Neuropeptide F

Neuropeptide F (NPF) is known to regulate feeding, sleep, and the circadian clock (He et al., 2013; Chung et al., 2017; Fadda et al., 2019). By a genetic screen with various neuropeptide mutants of *D. melanogaster*, it was found that a genetic null mutant of *NPF* does not induce an increase in female GSCs after mating (Ameku et al., 2018). In addition, the ovarian somatic cell-specific knockdown of NPF receptor phenocopies *NPF* genetic null mutant females, suggesting that NPF is received by the GSC niche cells through the NPF receptor. Additionally, the increase in the number of female GSCs can be evoked by injecting an artificially synthesized NPF peptide into virgin females or by *ex vivo* culture of virgin ovaries with NPF-supplemented medium. Moreover, NPF induces an increase in pMad intensity in female GSCs. Of note, Dpp signaling in the niche cells not only regulates GSC differentiation but also enhances GSC division (Xie and Spradling, 1998). Therefore, these results indicate that NPF is a humoral factor that directly acts on the GSC niche and is both necessary and sufficient to induce female GSC increase through the niche signal.

It is well established that *NPF* is expressed in the central brain and enteroendocrine cells (EECs) of the midgut (Chung et al., 2017; Guo et al., 2019). Therefore, the next question to be addressed was which NPF-producing tissue is responsible for mating-induced increase in number of female GSCs in *D. melanogaster* females – the brain or the midgut. Unexpectedly, the mating-induced increase in female GSC still occurs in brain-specific *NPF* knockdown animals, whereas *NPF* knockdown specifically in enteroendocrine cells (EECs) impairs mating-induced increase in female GSCs (Ameku et al., 2018). These results demonstrate that EEC-derived NPF contributes to an increase in female GSC after mating. Furthermore, activation of SP signaling promotes NPF release from EECs. The released NPF is received by ovarian somatic cells, where it enhances niche signaling from cap cells to female GSCs and induces an increase in the number of GSCs (Figure 2B). Although many genetic studies have exclusively focused on the brain NPF function, Ameku et al. (2018) is the first to demonstrate the crucial role of EEC-derived NPF in insect physiology. This is also the first example of interorgan communication in which gut hormones act on the ovary.

Neuropeptide F belongs to an evolutionary conserved neuropeptide Y (NPY)/NPF-type family of neuropeptides (Elphick and Mirabeau, 2014). This family also contains several mammalian neuropeptides, such as NPY and peptide YY (PYY). In particular, PYY is a well-known enteroendocrine hormone that acts on the hypothalamus and suppresses food intake (Schalla et al., 2021), whereas the precise function of PYY in maintenance or proliferation of stem cells in mammals has not yet been elucidated.

### Regulation of Germline Stem Cells by Octopamine

Another humoral factor is the insect neurotransmitter octopamine (OA), an analog of mammalian noradrenaline (Roeder, 2004; White et al., 2021b). There are a number of octopaminergic neurons in the brain of *D. melanogaster*, a particular subset of neurons (*dsx^+^ Tdc2* neurons) that project directly from the abdominal ganglion to the ovary. In addition, *dsx^+^ Tdc2* neurons are responsible for the regulation of egg-laying behavior (Monastirioti, 2003; Rezával et al., 2014; Deady and Sun, 2015; White et al., 2021b). Forced activation of these neurons results in an increase in the number of GSCs even in the virgin state (Yoshinari et al., 2020). Conversely, inactivation of *dsx^+^ Tdc2* neurons suppresses the mating-induced increase in female GSCs. Furthermore, *dsx^+^ Tdc2* neurons have synaptic contacts with SPSNs, which convey mating stimuli to *dsx^+^ Tdc2* neurons. After receiving the mating stimulus, *dsx^+^ Tdc2* neurons release OA, which is received by ovarian somatic cells through the G-protein coupled receptor called octopamine receptor in the mushroom body (Oamb). Liganded Oamb enhances Dpp signaling in female GSCs (Figure 2B). Thus, Yoshinari et al. (2020) revealed the neuroanatomical basis of the signal relay that transmits mating stimuli to female GSCs via neurons.

It was also observed that calcium ions (Ca^2+^) and matrix metalloproteinase 2 (Mmp2) are involved downstream of Oamb (Yoshinari et al., 2020). Consistent with previous studies showing that Oamb utilizes Ca^2+^ as a second messenger, the intracellular Ca^2+^ level in ovarian somatic cells are elevated by OA administration in an *ex vivo* culture system (Lee et al., 2009; Yoshinari et al., 2020). Downstream of Ca^2+^, Mmp2 acts on mating-induced female GSC proliferation. This model is supported by the observation that mating-induced female GSC proliferation is suppressed when the function of Mmp2 in ovarian somatic cells is inhibited (Yoshinari et al., 2020). Moreover, suppression of Mmp2 function in the ovary does not induce increase in female GSCs even in the presence of OA in *ex vivo* cultures. These results indicate that the activity of Mmp2 in ovarian somatic cells is required to induce post-mating increase in female GSCs, and that Mmp2 functions downstream of OA.

It has been reported that OA and Oamb are also essential for follicle rupture in the ovaries of *D. melanogaster* (Deady and Sun, 2015; Knapp and Sun, 2017). In this process, Mmp2 in follicle cells cleaves and downregulates collagen IV, also known as Viking (Vkg), a major component of the basement membrane (Wang et al., 2008; Deady et al., 2017). However, there was no significant change in the Vkg signal in the niche region in *Mmp2* knockdown in the germarium (Yoshinari et al., 2020). Currently, the substrate of Mmp2 in the GSC niche is unclear.

Octopamine is a neurotransmitter, analogous to mammalian noradrenaline/norepinephrine (Bauknecht and Jékely, 2017). Interestingly, noradrenergic neurons, which directly affect the bone marrow, also regulate the remodeling of hematopoietic stem cells niche (Méndez-Ferrer et al., 2008, 2010; Ho et al., 2019). Therefore, the noradrenergic nerve-dependent modulation of stem cell homeostasis may be conserved across animal species.

### Regulation of Germline Stem Cells by Ecdysteroids

In mammals, steroid hormones function as sex hormones and regulate the growth and physiological functions of reproductive organs during puberty and pregnancy (Zubeldia-Brenner et al., 2016). The major steroid hormones in insects are ecdysteroids, including the active ecdysteroid 20-hydroxyecdysone (20E), which are known to control molting and metamorphosis during larval development (Yamanaka and Okamoto, 2020; Kamiyama and Niwa, 2021a,b; Kannangara et al., 2021; Pan et al., 2021). Moreover, not only in larvae, but ecdysteroid biosynthesis and signaling are also active in adult insects, and are intricately involved in diverse processes, including oogenesis, stress resistance, longevity, and neuronal activity (Uryu et al., 2015). In particular, ecdysteroids are biosynthesized in the ovary where Halloween genes encoding ecdysteroidogenic enzymes are expressed (Niwa and Niwa, 2014; Uryu et al., 2015; Ameku and Niwa, 2016). Also, ecdysteroid signaling in the ovary regulates a wide range of oogenesis processes, including the maintenance of female GSC numbers (Ables and Drummond-Barbosa, 2010; König et al., 2011; Morris and Spradling, 2012; Ables et al., 2015; Ahmed et al., 2020; Finger et al., 2021). Additionally, 20E acts on both GSCs and the GSC niche cells to regulate the maintenance of female GSC numbers (Ables and Drummond-Barbosa, 2010; König et al., 2011).

A previous study reported the post-mating increase in ecdysteroid titer in the entire female body of *D. melanogaster* (Harshman et al., 1999; Ameku and Niwa, 2016). This elevation of ecdsyteroid titer after mating is apparently associated with several post-mating responses, such as increased food intake (Carvalho et al., 2006; Hadjieconomou et al., 2020), gut remodeling (Ahmed et al., 2020), and female GSCs increase (Ameku and Niwa, 2016; Ameku et al., 2017). Knockdown of *ecdysone receptor* (*EcR*) or ecdysteroid biosynthetic enzyme genes in ovarian somatic cells impairs the mating-dependent increase in female GSCs (Ameku and Niwa, 2016). Although it has not examined if ecdysteroids directly act on female GSCs to modulate their response to mating, these results clearly suggest that the action of ecdysteroids, at least in the ovarian somatic cells, is indispensable for the mating-induced increase in female GSCs (Figure 2B). On the other hand, oral administration of 20E does not induce increase in female GSCs in virgin *D. melanogaster*, suggesting that ecdysteroids alone are not adequate for increasing female GSCs.

Moreover, the correlation between ecdysteroids, NPF, and OA in regulating the number of female GSCs has also been explored to some extent. Ecdysteroid signaling is required for both NPF-dependent and OA-dependent increase in post-mating female GSC. Also, it has been reported that even in the presence of NPF or OA in *ex vivo* culture, if the *EcR* or ecdysteroid biosynthetic enzyme function is inhibited in the ovary, there is no increase in female GSC (Ameku et al., 2018; Yoshinari et al., 2020). This observation indicates that the ecdysteroid signaling influences NPF and OA sensitivity of the GSC niche. Notably, ecdysteroids are also involved in chromatin remodeling in the ovary (Ables and Drummond-Barbosa, 2010). Therefore, it may be hypothesized that ecdysteroid-mediated chromatin remodeling is an essential prerequisite for NPF- and OA-mediated post-mating increase in female GSCs.

Moreover, in the follicle rupture process, ecdysteroid signaling cooperates with the OA-Oamb-Ca^2+^-Mmp2 pathway (Deady and Sun, 2015). Therefore, the OA- and ecdysteroid-mediated synergistic regulation of mating-induced increase in female GSCs and follicle rupture are remarkably similar.

## Are Insulin-Like Peptides and Juvenile Hormones in *Drosophila Melanogaster* Involved in Mating-Induced Increase in Female Germline Stem Cells?

Interestingly, the first-identified hormones to influence female GSC numbers are *Drosophila* insulin-like peptides (DILPs) (LaFever and Drummond-Barbosa, 2005; Hsu et al., 2008; Hsu and Drummond-Barbosa, 2009, 2011). DILPs are one of the extensively studied nutrient-dependent signals in *D. melanogaster* and are structurally similar to vertebrate insulin (Kannan and Fridell, 2013; Koyama et al., 2020). DILP secretion from insulin-producing cells in the brain is strictly regulated in response to nutrient availability. Also, DILPs not only directly regulate the number of eggs laid, but also the GSC division, cyst growth, and vitellogenesis (LaFever and Drummond-Barbosa, 2005; Hsu et al., 2008). In the germarium, the DILP receptor called the insulin receptor (InR) is expressed in female GSCs and their surrounding somatic cells, including cap cells and follicle cells (Hsu and Drummond-Barbosa, 2009, 2011). The number of female GSCs and cap cells increases in high-protein diet conditions through the regulation of InR-dependent signaling (Hsu and Drummond-Barbosa, 2009). On the other hand, female *D. melanogaster* that are fed a low-protein diet show reduced InR signaling, leading to a decrease in the number of female GSCs and cap cells. In addition, regardless of nutritional conditions, the loss of *InR* function in female *D. melanogaster* leads to significantly low mitotic activity of female GSCs, similar to that of oligotrophs. Furthermore, the DILP signaling-mediated regulation of female GSC division is G2-phase specific (Kao et al., 2015).

It has also been demonstrated that DILPs not only reflect the nutrition condition but also the aging status of female GSCs. With aging, the number of cap cells and female GSCs decreases along with the reduction in DILP signaling in the ovary (Hsu and Drummond-Barbosa, 2009; Kao et al., 2015). In addition, suppression of *InR* function accelerates the reduction in cap cell and female GSC numbers during aging (Hsu and Drummond-Barbosa, 2009). Therefore, in the regulation of female GSCs via the niche, DILP-InR signaling is an essential endocrine pathway to effectively detect minute changes in nutritional conditions and age-related status.

We initially hypothesized that DILPs-InR signaling might be involved in the mating-induced increase in female GSCs. However, even when *InR* is knocked down by RNAi in the ovarian somatic cells, the mating-induced increase in female GSCs remains unperturbed (Ameku and Niwa, 2016). In contrast to the fact that the number of cap cells are regulated by DILP-InR signaling, mating does not affect cap cell numbers (Ameku and Niwa, 2016). Therefore, while it still seems plausible that DILP-InR signaling in germ cells affect female GSCs after mating, the data so far does not imply a direct contribution of DILP-InR signaling in the increase in the number of female GSCs.

A recent study reported that the female GSC numbers are regulated by another important class of insect hormones, namely the juvenile hormones (JHs) (Luo et al., 2020). JHs are sesquiterpenoids synthesized in the *corpora allata* (Jindra et al., 2013; Santos et al., 2019; Kurogi et al., 2021). JH binds to two JH receptors, Methoprene-tolerant (Met) and Germ cell-expressed bHLH-PAS (Gce) (Jindra et al., 2013). Double knockdown of *Met* and *Gce* in cap cells and terminal filament cells reduces the number of female GSCs (Luo et al., 2020). This also validated in animals where JH biosynthetic enzyme (JH acid *O*-methyltransferase) is knocked down (Luo et al., 2020). Notably, the synthesis of JHs in *ex vivo* cultured *corpora allata* is stimulated by synthetic SP (Moshitzky et al., 1996). Moreover, JH concentration in hemolymphs increases after mating and acts as a mating signal for intestinal remodeling (Reiff et al., 2015). These observations suggest that JH mediates mating-induced increase in number of female GSCs. However, a mating-induced increase in female GSCs is still observed even when *Met* or *Gce* is knocked down in ovarian somatic cells alone (Ameku and Niwa, 2016). In addition, feeding virgin female *D. melanogaster* with the JH analog, methoprene, does not induce an increase in GSCs (Ameku and Niwa, 2016). Therefore, the data so far imply that JH signaling may not be solely responsible for the increase in female GSCs after mating. Hence, further studies are warranted to elucidate the role of JHs in mating-induced increase in female GSCs.

## Remaining Questions

### How Does the Regulation of Germline Stem Cell Division Lead to Mating-Induced Increase in Female Germline Stems?

Stem cells can divide symmetrically and asymmetrically, which yield progeny stem cells and more differentiated cells, respectively. It is speculated that NPF, OA, and ecdysteroids promote symmetric, rather than asymmetric cell division of female GSCs, causing to mating-inducible increase in number of female GSCs. Previous research also suggest that the Dpp niche signaling is essential for the mating-induced increase in female GSC number (Ameku and Niwa, 2016; Ameku et al., 2018; Yoshinari et al., 2020). However, it is yet unknown how the humoral factors accelerate cell divisions and regulate the polarity of female GSC cell division by modulating the niche signal. In addition, it has not been elucidated whether the increase of female GSCs after mating is also implemented by the increase in dedifferentiation of the cysts (Herrera and Bach, 2018). Therefore, in future, *ex vivo* live-imaging techniques for *D. melanogaster* ovaries (Parton et al., 2010; He et al., 2011; Morris and Spradling, 2011; Cetera et al., 2016; Reilein et al., 2018; Villa-Fombuena et al., 2021), might be a powerful tool to resolve this debate. In particular, a recent report has optimized the experimental conditions that allow prolonged observation of *ex vivo* cultured germaria, and has succeeded in capturing the live images of both symmetric and asymmetric GSC division by visualizing the spectrosome cycle with GFP-fused Par-1 protein (Villa-Fombuena et al., 2021).

### How Do Multiple Humoral Factors Induce Increase in Female Germline Stem Cells?

Although mating-induced increase in female GSCs appears to be very simple, the neuroendocrine mechanisms regulating this phenomenon are rather complicated. At least three humoral factors, ecdysteroids, NPF, and OA, are involved in the post-mating process, and the downstream signaling pathways of these humoral factors are different. Although active ecdysteroids bind to their receptor EcR and play a role in transcriptional regulation, cyclic AMP and Ca^2+^ function as second messengers downstream of NPF and OA, respectively (Figure 2B; Garczynski et al., 2002; Lee et al., 2009). Various questions still remain unaddressed such as, how does the signaling downstream of NPF and OA work concurrently? How does each of these signaling pathways independently play a role in cell division? What are the precise functions of NPF and OA signaling in cellular proliferation? Although the answers to these questions are obscure, it is believed that the integration of these signals may ultimately influence the quality of cell division and differentiation by enhancing Dpp signaling. Moreover, it is established that NPF and OA are received by ovarian somatic cells resulting in Dpp signaling enhancement (Ameku et al., 2018; Yoshinari et al., 2020). However, precisely which somatic cell types of the ovary express their receptors and contribute to the modulation of Dpp signaling remains elusive. Therefore, it is intriguing to elucidate how humoral factors and their downstream signals interact with each other to regulate increase in female GSCs after mating. Such interaction between these diverse factors is expected to precisely control the GSC increase in response to changes in the environment.

### Do Nutrition and Mating Stimuli Interact With Each Other to Influence Germline Stem Cells?

Mating and nutritional status affect each other. In general, mating behavior is controlled by nutritional conditions. For example, in *D. melanogaster*, dietary yeast increases the female sexual receptivity through the interaction of the yeast proteins with its odorous fermentation product, acetic acid (Gorter et al., 2016). Moreover, nutritional requirements and nutrition-related physiological status change after mating. Consistent with this finding, it is observed that in female *D. melanogaster*, mating stimulates the food intake (Carvalho et al., 2006; Itskov et al., 2014), food preference (Ribeiro and Dickson, 2010; Vargas et al., 2010; Walker et al., 2015; Corrales-Carvajal et al., 2016; Leitão-Gonçalves et al., 2017; Carvalho-Santos et al., 2020), starvation resistance (Rush et al., 2007; Jang and Lee, 2015), and midgut morphology and physiology (Reiff et al., 2015; White et al., 2021a). As mentioned above, female GSCs are closely related to nutritional conditions. Therefore, it is possible that changes in nutritional status affect the increase in female GSCs after mating, and thus their post-mating response.

Recent studies have revealed that the release of NPF in EECs is induced not only by mating but is also sugar-dependent, and has a function similar to that of incretins in mammals (Yoshinari et al., 2021). When *D. melanogaster* is reared on food that excludes sugar, intestinal NPF secretion is suppressed, and NPF release is induced by sugar refeeding. When intestinal NPF function is suppressed, *D. melanogaster* exhibits various metabolic dysfunction such as, lipodystrophy, hyperphagia, and hypoglycemia (Yoshinari et al., 2021), which is partly reminiscent of the function of mammalian PYY (Schalla et al., 2021). This sugar-dependent NPF release is partly mediated by the sugar transporter Sut1, which is expressed in NPF-positive EECs. The released NPF is accepted by NPF receptors expressed in insulin-producing cells and *corpora cardiaca* (CC) (Yoshinari et al., 2021). It is clear that insulin-producing cells are responsible for the production and secretion of DILPs, which are essential for the regulation of hemolymph glucose levels and fat storage (Nässel et al., 2013). On the other hand, CC cells are responsible for the release of adipokinetic hormone (AKH), the insect counterpart of vertebrate glucagon. Under energy-demanding conditions, AKH is released from the CC into the hemolymph, where it is accepted by the AKH receptor (AKHR), expressed in the fat body (Grönke et al., 2007; Bharucha et al., 2008; Gáliková et al., 2015). The fat body is the main lipid storage organ in insects. AKH-AKHR signaling induces the degradation of triacylglycerol stored in the fat body, resulting in the production and release of diacylglycerides (Grönke et al., 2007).

A study reported that NPF-NPFR signaling promotes the secretion of DILP, and inhibits the production of AKH and ultimately lipid assimilation (Yoshinari et al., 2021). These results suggest that EEC-derived NPF regulates sugar-dependent metabolism via glucagon-like and insulin-like hormones in insects and mammals. Thus, NPF receives not only mating information, but also nutrition information. It would be interesting to investigate how EECs integrate mating stimuli with nutritional information to regulate the release of NPF.

It is also possible that mating-induced increase in female GSCs also requires tissues not yet identified in the studies described above, such as the fat body. A recent study has implied that the post-mating response is also present in the fat body, as triacylglycerol levels increase after mating (Reiff et al., 2015). After mating, a large amount of energy is required to activate oogenesis (Carvalho et al., 2006). With this energy demand, the fat body may undergo remodeling to facilitate the smooth storage and mobilization of energy. The mechanism of nutrient-dependent regulation of female GSCs is also closely related to the fat body (Armstrong et al., 2014; Matsuoka et al., 2017; Weaver and Drummond-Barbosa, 2018). Suppression of amino acid transporter function in the fat body results in a decrease in the number of oocytes or female GSCs (Armstrong et al., 2014). It has also been shown that the fat body provides Vkg/collagen IV that is important for the formation of the GSC niche (Weaver and Drummond-Barbosa, 2018). Vkg from the fat body is also found to be embedded in the GSC niche and is important for maintaining normal levels of E-cadherin, which tightly adheres female GSCs to the niche. While we failed to see the involvement of Vkg in mating-induced increase in female GSCs in our static imaging, it will still be interesting to examine whether dynamics of Vkg deposition and deconstruction in the GSC niche changes before and after mating.

### Is the Metabolic State of Germline Stem Cells Altered After Mating?

Recent discoveries have revealed that metabolites regulate stem cell fate by regulating intracellular signaling and enzyme activity (Shyh-Chang and Ng, 2017; Harvey et al., 2019). For example, the genes associated with glycolysis and the citric acid cycle are expressed at very low levels in young female GSCs (Teixeira et al., 2015). In particular, the transcript levels of genes encoding aldolase and oxoglutarate dehydrogenase are hardly detectable. In addition, mitochondrial cristae are immature in female GSCs, but they mature in a mitochondrial ATP synthase-dependent manner when female GSCs differentiate (Teixeira et al., 2015). Moreover, female GSC differentiation and maintenance are unaffected even in knock-down animals of each of the 13 nuclear-encoded ATP synthase subunits. These observations imply that the ATP in female GSCs is not supplied by internal oxidative metabolism, but rather from other sources that are currently unknown.

Another example is the pentose phosphate pathway, which is activated when female GSCs transform to a differentiated stage (Carvalho-Santos et al., 2020). Although the pentose phosphate pathway is not active in female GSCs, its activity is increased in germ cells at the posterior end of the germarium. Inhibiting the function of the pentose phosphate pathway or removing germ cells reduces the amount of sugar consumed and the feeding preference for sugar. This suggests that germ cells themselves change their sugar intake and preference in accordance with the demand for sugar in differentiated germ cells. It would be interesting to investigate whether this metabolism is affected by various factors, such as mating and nutrition.

### Do Cryptic Genetic or Environmental Factors Induce Increase in Post-mating Increase in Germline Stem Cells?

Lastly, although our group has reported an increase in mating-induced female GSCs, other research groups have reported that post-mating increase in female GSCs has not been observed in their laboratories (Kao et al., 2015; Malpe and Schulz, 2020). Currently, we do not know the particular conditions that influence such discrepancies. This discrepancy may be due to differences in the genetic backgrounds of *D. melanogaster* strains, the number of mating attempts, food conditions, or fly feeding conditions. For example, in the study conducted by Kao et al. (2015), the authors housed 10 females and 5 males in one vial containing a standard cornmeal-dry yeast-sugar-agarose fly food with wet yeast paste. However, in our experiments, we usually house 10–20 females with the equal number of males in one vial containing the standard cornmeal-dry yeast-sugar-agarose fly food without wet yeast paste. Authors Malpe and Schulz (2020) conducted their mating assay at 29°C with yeast paste and apple juice as diet, while our group usually conducts the mating assay at 25°C with a standard cornmeal-dry yeast-sugar-agarose fly food. Additionally, from the currently available literature, we cannot precisely compare the differences in diverse fly food recipes used in various laboratories. We expect that the elucidation of the causalities would be a meaningful approach to unravel the molecular and neuroendocrine mechanisms of mating-induced increase in female GSCs.

## Future Perspective

The number of GSCs is determined by sensing the internal and external environments of the individual. This regulatory mechanism has biological significance in that it prevents unnecessary energy consumption by ensuring that egg formation occurs only when necessary. Our future research direction is aimed at determining how mating is linked to internal and external status, such as aging and nutritional status; to alter niche signaling and regulation of GSCs. It will also be interesting to see whether such nutritional status, age, and egg-laying experience prevent post-mating responses other than the increase in female GSCs. A series of studies have also shown that crosstalk among different organs, such as the intestine, is important for the environment-dependent control of fertility. In other words, there is a need for a comprehensive understanding of how each organ responds to environmental changes and how this response affects other organs. The study of environmental responses via inter-organ crosstalk in *D. melanogaster* is a model case for elucidating the adaptability of an organism, which changes in various ways, depending on its status within an individual.

Lastly, we would like to note that female GSCs have scarcely been studied in other insects than *D. melanogaster* (Niwa and Kai, 2020). In most insects, even female GSCs themselves are yet to be identified cytologically. On the other hand, many kinds of post-mating responses are observed in most insects (Avila et al., 2010). Fueled by the advances in understanding post-mating increase in GSCs in *D. melanogaster*, we hope that studies using other insects will also be stimulated and developed in the future.

## Author Contributions

RH and RN wrote and revised the manuscript and approved the submitted version.

## Conflict of Interest

The authors declare that the research was conducted in the absence of any commercial or financial relationships that could be construed as a potential conflict of interest.

## Publisher’s Note

All claims expressed in this article are solely those of the authors and do not necessarily represent those of their affiliated organizations, or those of the publisher, the editors and the reviewers. Any product that may be evaluated in this article, or claim that may be made by its manufacturer, is not guaranteed or endorsed by the publisher.
